# Procyanidin Capsules Combat ALF by Restoring Mitochondrial Homeostasis and Inhibiting Necroptosis via the PGAM5/DRP1/PINK1 Pathway

**DOI:** 10.1002/advs.202508742

**Published:** 2025-12-03

**Authors:** Qing Shi, Minmin Wu, Jinwei Zhong, Chao Chen, Zhuang Huang, Jingxuan Peng, Dong Yang, Xingjie Zan, Zhengfei Wang

**Affiliations:** ^1^ Department of Gastroenterology The First Affiliated Hospital of Wenzhou Medical University Wenzhou 325035 China; ^2^ Zhejiang Key Laboratory of Intelligent Cancer Biomarker Discovery and Translation The First Affiliated Hospital of Wenzhou Medical University Wenzhou 325035 China; ^3^ Wenzhou Medical University Wenzhou 325035 China; ^4^ Wenzhou Institute Wenzhou Key Laboratory of Perioperative Medicine University of Chinese Academy of Sciences Wenzhou 325001 China; ^5^ The Quzhou Affiliated Hospital of Wenzhou Medical University Quzhou People's Hospital Quzhou Zhejiang 324000 China

**Keywords:** acute liver failure, mitochondrial homeostasis, necroptosis, PGAM5/DRP1/PINK1, procyanidin capsules

## Abstract

Acute liver failure (ALF) is a life‐threatening, multifactorial condition characterized by rapid progression, extensive hepatocellular necrosis, and high mortality rates. Current therapeutic options, including artificial liver support systems (ALSS) and liver transplantation, are limited by high costs, donor shortages, and insufficient efficacy. Mitochondrial dysfunction and necrotic cell death play central roles in both acute and chronic liver injury; however, their contribution to ALF remains poorly understood. In this study, self‐assembled procyanidin capsules (PC‐Ca) are developed with sustained antioxidant and anti‐inflammatory properties that selectively accumulate in the liver of an ALF model. These findings demonstrate that PC‐Ca significantly improves survival rates and more effectively mitigates liver injury, inflammation, and necrosis in thioacetamide (TAA)‐induced ALF in mice and rabbits than the standard clinical agent, N‐acetylcysteine (NAC). This protective effect is mediated through enhanced oxidative stress defense via activation of the KEAP1‐NRF2 axis and inhibition of necroptosis via the RIPK1/RIPK3/MLKL pathway. In addition, PC‐Ca preserves mitochondrial morphology and function via the PGAM5/DRP1/PINK1 pathway, offering hepatoprotection. These findings suggest that PC‐Ca represents a promising therapeutic strategy for ALF, with the modulation of mitochondrial homeostasis offering valuable insights for the development of next‐generation pharmacological interventions.

## Graphical abstract

A biomimetic self‐assembly strategy, Procyanidin Capsules (PC‐Ca), has been developed, which has great stability, bioavailability, and liver‐targeting efficacy and modulates the KEAP1‐NRF2 axis to inhibit ROS formation and necroptosis, regulate mitochondrial homeostasis through the PGAM5/DRP1/PINK1 signaling pathway in thioacetamide (TAA)‐induced ALF models.

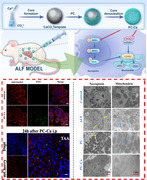

## 1 Introduction

Acute liver failure (ALF) is a life‐threatening clinical condition characterized by rapid hepatocyte necrosis, severe dysfunction, and progressive development of hepatic encephalopathy, with mortality rates often exceeding 60% in the absence of liver transplantation.^[^ ^1^ ^]^ Although liver transplantation remains the gold standard treatment, its application is limited by challenges such as organ shortages, complex surgical procedures, and lifelong requirements for immunosuppressive therapy.^[^ ^2^, ^3^ ^]^ Emerging therapeutic alternatives, including bioartificial liver support systems and stem cell‐based therapies, are hindered by significant technical barriers and resource demands, which limit their widespread clinical adoption.^[^ ^4^ ^]^ Pharmacological interventions, particularly N‐acetylcysteine (NAC), have demonstrated partial efficacy in acetaminophen‐induced ALF, improving the transplant‐free survival rates from 27% to 58%.^[^ ^5^ ^]^ However, the narrow therapeutic window of NAC and incomplete understanding of its underlying mechanisms underscore the pressing need for novel therapeutic approaches targeting the fundamental pathophysiology of ALF.^[^ ^6^ ^]^

The core pathological feature of ALF is a progressive and sustained cycle of mitochondrial dysfunction driven by various stressors, including infection, metabolic disturbances, and tissue injury. These stressors lead to the overproduction of TNF‐α and reactive oxygen species (ROS), inducing oxidative stress in hepatocytes.^[^ ^7^, ^8^, ^9^, ^10^, ^11^ ^]^ Oxidative stress disrupts the function of the electron transport chain, damages mitochondrial membranes and DNA, and activates the formation of necroptosis signaling complexes (RIPK1/RIPK3/MLKL).^[^ ^12^ ^]^ These necroptosis complexes exacerbate mitochondrial dysfunction by promoting the activation of mitochondrial phosphatase PGAM5, which impairs PINK1‐mediated mitochondrial autophagy.^[^ ^13^, ^14^ ^]^ Furthermore, the dephosphorylation of PGAM5 enhances its interaction with DRP1, leading to excessive mitochondrial fission, increased ROS production, and mitochondrial damage.^[^ ^15^, ^16^ ^]^ This results in a feedback loop of “oxidative stress–mitochondrial dysfunction–necroptosis,”^[^ ^17^, ^18^ ^]^ culminating in irreversible hepatocyte necrosis, mitochondrial permeabilization, ionic imbalance, cellular swelling, and rupture.

Recent studies have highlighted the therapeutic potential of targeting the formation and remodeling of necroptosis complexes as well as modulating mitochondrial function to ameliorate liver injury in ALF. Genetic ablation of RIPK3/MLKL in models of steatohepatitis and acetaminophen‐induced liver injury significantly reduced hepatocyte necroptosis and accelerated liver recovery.^[^ ^19^, ^20^ ^]^ Inhibition of RIPK1 to prevent necroptosis has also been shown to reduce the viral load in hepatitis B virus (HBV) infections, mitigating liver damage.^[^ ^21^ ^]^ Additionally, modulation of mitochondrial dysfunction in Kupffer cells has been reported to alleviate necroptosis in metabolic dysfunction‐associated steatosis liver disease (MASLD).^[^ ^22^ ^]^ However, increased mitochondrial ROS production accelerates necroptosis in hepatocellular carcinoma (HCC) and colorectal cancer (CRC) models, indicating the delicate balance required to target mitochondrial dysfunction.^[^ ^23^, ^24^ ^]^ These findings highlight the promising therapeutic potential of necroptosis modulation in liver injury and related malignancies. However, strategies specifically targeting mitochondrial dysfunction and necroptosis in ALF remain underexplored.

**Figure 1:**
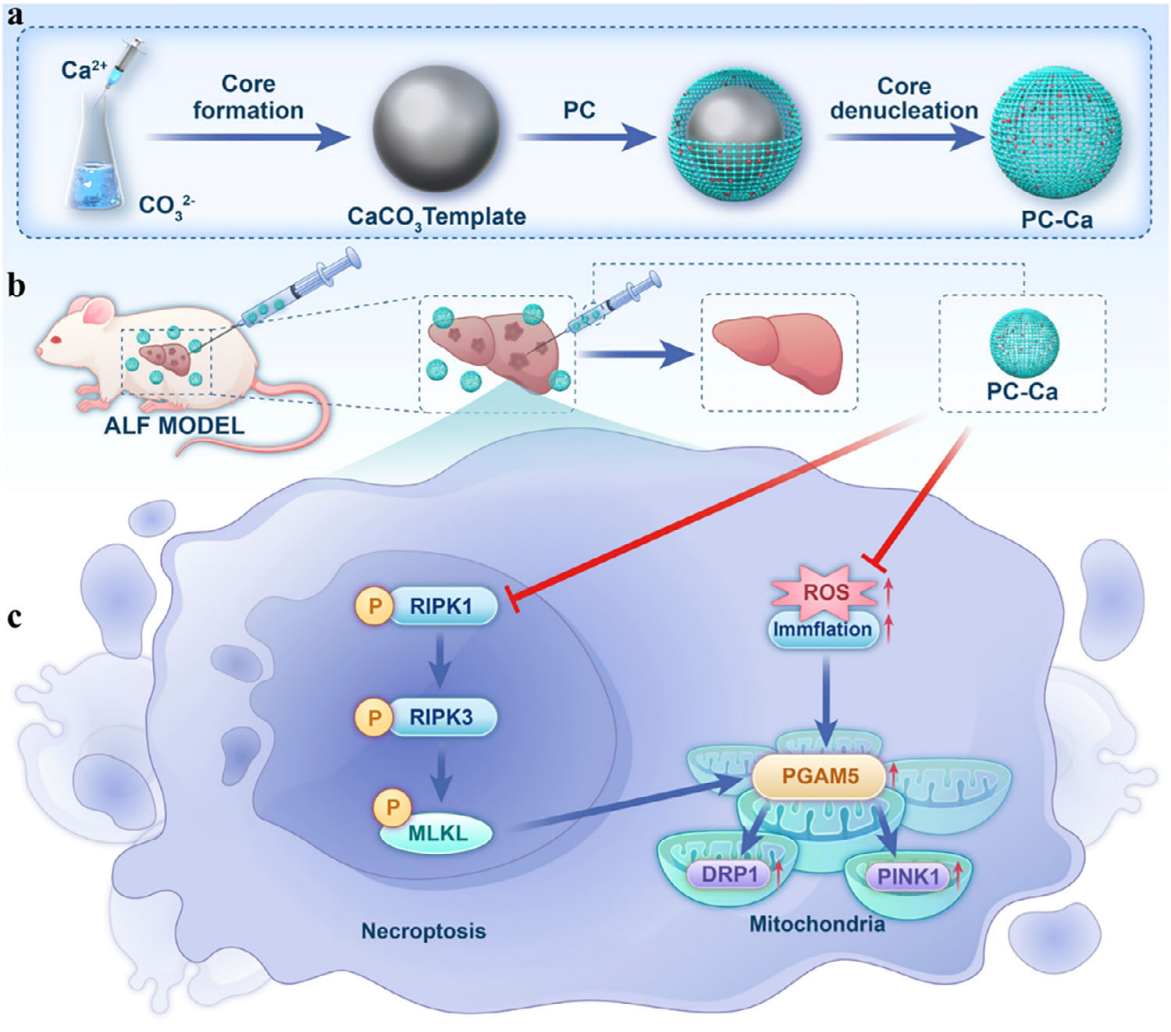
The synthesis and mechanism diagram of PC‐Ca after intraperitoneal injection in TAA‐Induced ALF mice in vivo. a) The synthesis method of polycarbonate doped with calcium carbonate; b,c) The mechanism diagram of PC‐Ca after intraperitoneal injection in TAA‐induced ALF mice. PC‐Ca significantly inhibited the phosphorylation of RIPK1/RIPK3/MLKL and the expression of PGAM5/DRP1/PINK1 to protect mitochondrial morphology and homeostasis against necroptosis to alleviate ALF.

Polyphenols, such as procyanidin (PC), are potent antioxidants that demonstrate ROS‐scavenging activity through hydrogen atom transfer. Despite their promising therapeutic properties, their clinical application in liver diseases remains limited owing to pharmacokinetic challenges, including poor solubility, low bioavailability, and insufficient hepatic accumulation.^[^ ^25^, ^26^, ^27^, ^28^, ^29^ ^]^ To overcome these limitations, we developed PC capsules (PC‐Ca) using a biomimetic self‐assembly strategy (**Figure** **1**a). This formulation preserves the natural structure and bioactivity of PC and significantly improves its stability, bioavailability, and liver‐targeting efficacy. In preclinical models of ALF, a single intraperitoneal injection of PC‐Ca demonstrated better therapeutic efficacy than NAC, improving survival rates and liver function and mitigating inflammation and oxidative stress. Mechanistic studies revealed that PC‐Ca modulates the KEAP1‐NRF2 axis to inhibit ROS formation while also regulating mitochondrial homeostasis through the PGAM5/DRP1/PINK1 signaling pathway. This dual mechanism reduced mitochondrial ROS production and alleviated necrosis and inflammation in thioacetamide (TAA)‐induced ALF models (Figure 1b,c). The multifunctional nature of this therapeutic approach provides a promising paradigm for the treatment of ALF by targeting both oxidative stress and mitochondrial dysfunction using a unified pharmacological strategy.

## 2 Results

### 2.1 Preparation, Characterization, and ROS/RNS Scavenging Ability of PC Capsules

Confocal Laser Scanning Microscopy (CLSM) bright‐field and green fluorescence imaging revealed the internal cavity structure of PC‐Ca and its good distribution characteristics in solution, with the appearance of green circles confirming the formation of capsules (Figure S1a,b, Supporting Information). PC‐Ca exhibited a uniform vesicular shape under Scanning Electron Microscopy (SEM; Figure S1c, Supporting Information). The Fourier Transform infrared spectroscopy (FTIR) spectrum of the PC‐Ca showed absorption peaks similar to those of PC (Figure S2a, Supporting Information), except for the peak corresponding to hydrogen bonding, which shifted to 3400 cm^−1^, due to the formation of hydrogen bonds between the phenolic hydroxyl groups of PC.^[^ ^30^ ^]^ X‐ray Photoelectron Spectroscopy (XPS) results indicated that the composition of PC‐Ca after the EDTA/HCL template removal contained only carbon and oxygen (Figure S2b, Supporting Information), indicating the absence of toxic elements. Stability analysis was conducted by placing the capsules in pure water, phosphate‐buffered saline (PBS), 0.9% sodium chloride (NaCl), and Dulbecco's modified Eagle's medium (DMEM) for 2 months. In all the cases, the capsules maintained their morphology (Figure S2c, Supporting Information), similar to that shown in Figure S1c (Supporting Information). Formulating polyphenols into microparticles helps protect the phenolic hydroxyl groups from oxidation, thereby slowing the loss of antioxidant function and achieving long‐term antioxidant efficacy.^[^ ^31^ ^]^

To demonstrate the long‐term ROS and reactive nitrogen species (RNS)‐scavenging capabilities of the polyphenolic capsules, the capsules were stored in an oxygen environment at room temperature for 0 h and 1 month. Total antioxidant capacity, 1,1‐diphenyl‐2‐picryl‐hydrazyl radical (DPPH), and 2,2′‐azinobis‐ (3‐ethylbenzthiazoline‐6‐sulfonate) (ABTS) radical scavenging abilities were then tested, with results shown in Figure S3 (Supporting Information). To compare the antioxidant characteristics of Vitamin C (VC), PC, and PC‐Ca, the ROS and RNS scavenging rates were calculated after 1 month of storage. The total antioxidant capacity, H_2_O_2_ scavenging ability, O_2_^−^ scavenging ability, and DPPH scavenging ability of VC and PC significantly decreased after 1 month of storage (Figure S3a,b, Supporting Information). By contrast, the total antioxidant capacity, H_2_O_2_ scavenging ability, O2^−^ scavenging ability, and DPPH scavenging ability of PC‐Ca decreased only slightly (Figure S3a–c, Supporting Information) (red bars represent the effects after 1 month of storage). Furthermore, compared with PC, PC‐Ca exhibited a significantly stronger total antioxidant capacity, DPPH scavenging ability, and ABTS radical scavenging ability (*p*< 0.0001), both in the short term and after 1 month. This indicates that PC‐Ca has a better long‐term, stable ROS‐scavenging ability than PC solutions and VC, which is beneficial for the treatment of liver damage.

### 2.2 Biocompatibility, Biodistribution In Vivo

Prior to the in vivo study, we evaluated the blood compatibility of PC‐Ca using a hemolysis assay in vitro (Figure S4, Supporting Information). Different concentrations of PC‐Ca did not exhibit hemolytic effects on RBCs in vitro, confirming the blood compatibility of PC‐Ca. To further evaluate the systemic toxicity of PC‐Ca (100 mg kg^−1^) after intraperitoneal injection, hematoxylin and eosin (H&E) staining of the heart, liver, lung, spleen, and kidney showed no significant pathological changes in PC‐Ca‐treated mice (Figure S5, Supporting Information). Additionally, compared with the control group, in the PC‐Ca group, the levels of alanine aminotransferase (ALT), aspartate transaminase (AST), and blood platelets (PLT) were not significantly different (Figure S6, Supporting Information). These results indicate that PC‐Ca has no toxicity and has excellent biocompatibility both in vivo.

To evaluate the in vivo biodistribution of PC‐Ca, we intraperitoneally administered PC‐Ca labeled with mCherry（m‐Cherry‐PC‐Ca) to mice and monitored its accumulation in the liver using the IVIS Lumina II in vivo imaging system. As shown in Figure S7a (Supporting Information), after intraperitoneal injection of mCherry‐PC‐Ca in normal mice, the accumulation of PC‐Ca in vivo was mainly distributed around the intraperitoneal injection site, whereas in TAA‐induced ALF models, its fluorescence intensity was concentrated around the liver in response to acute hepatitis. Nevertheless, dissection of the major organs at 24 h revealed that mCherry‐PC‐Ca accumulated in the liver had a greater tendency than in other organs (Figure S7b, Supporting Information). Moreover, the distribution of mCherry‐PC‐Ca in liver tissue sections was observed using confocal laser scanning microscopy, as shown in Figure S7c (Supporting Information). The accumulation of mCherry‐PC‐Ca in the liver tissue of TAA‐induced ALF models was greater than that in normal mice.

### 2.3 Therapeutic Efficacy of TAA‐Induced Acute Liver Failure in Mice

**Figure 2:**
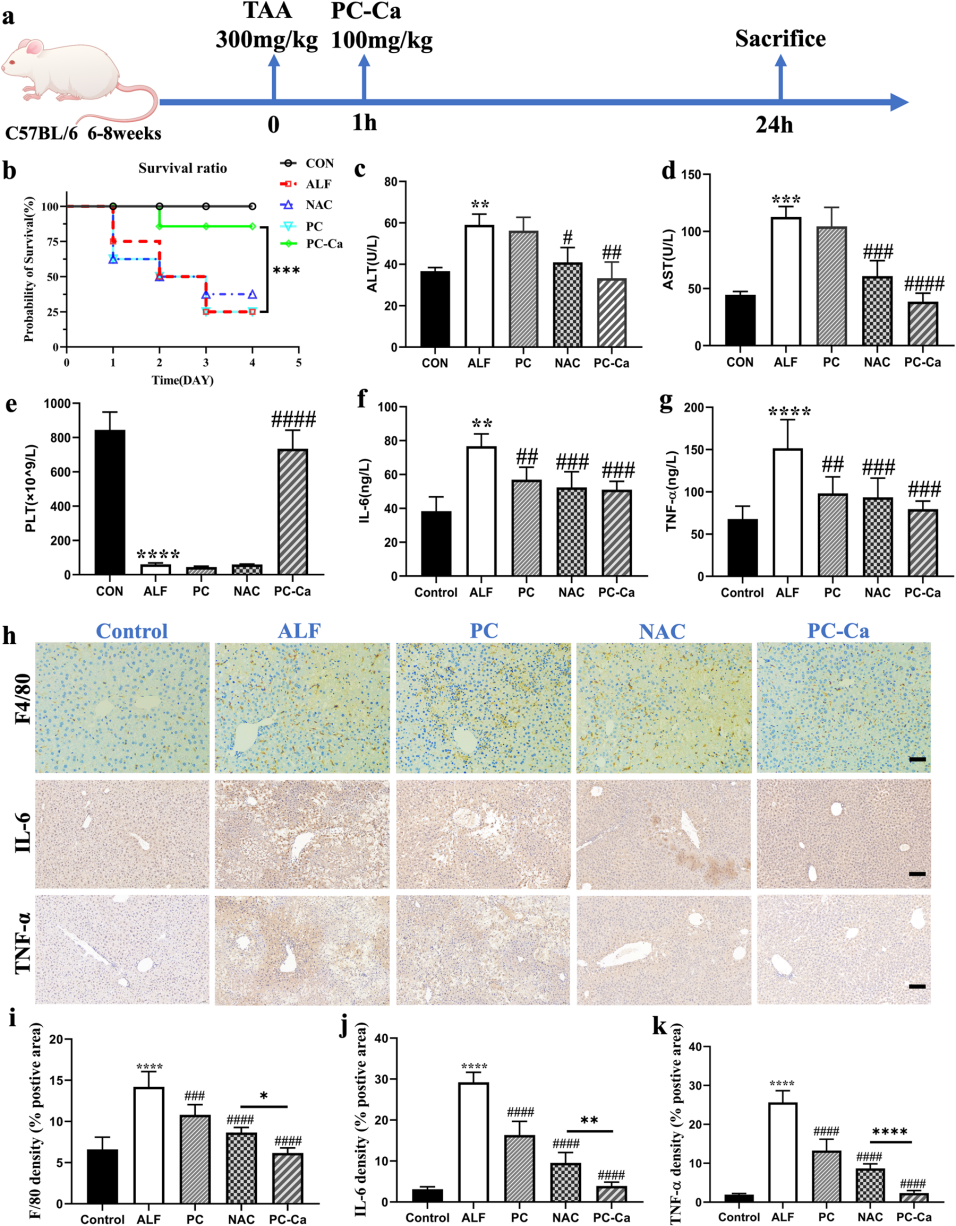
The therapeutic efficacy of PC‐Ca after intraperitoneal injection in TAA‐Induced Acute Liver Failure mice in vivo. a) The mice were injected intraperitoneally with 300 mg kg^−1^ TAA. After 1 h post‐modeling, the mice with TAA were injected intraperitoneally (i.p) with PC, NAC, and PC‐Ca (100 mg kg^−1^, respectively). After 24 h post‐modeling, they were sacrificed for subsequent experiments. b) Survival curves of mice with different treatments (n = 10, ^***^ *p*< 0.001). c–e) The levels of ALT, AST, and PLT at 24 h after TAA intoxication. f,g) The levels of inflammatory cytokines (IL‐6, TNF‐α) by ELISA at 24 h after TAA intoxication. h) Representative F4/80, IL‐6, and TNF‐α by immunohistochemistry (IHC) staining of liver tissues. Scale bar = 100 µm. i–k) Quantification of F4/80, IL‐6, and TNF‐α density of % positive area. ^*^ *p*< 0.05, ^**^ *p*< 0.01, ^***^ *p*< 0.001, ^****^ *p*< 0.0001 vs Control group, ^##^ *p*< 0.01, ^###^ *p*< 0.001, ^####^ *p*< 0.0001 vs ALF group).

The mice were intraperitoneally injected with 300 mg kg^−1^ TAA. At 1 h post‐modeling, TAA‐treated mice were injected intraperitoneally (i. p.) with NAC (100 mg kg^−1^) and PC‐Ca (100 mg kg^−1^). After 24 h, the mice were sacrificed for subsequent experiments (**Figure** **2**a). Since there is no specific treatment available for drug‐induced ALF resulting in multi‐organ failure and high mortality, the mice were guided to survive following the TAA challenge and subsequent supervision of PC‐Ca to explore its therapeutic efficacy. NAC was used as a positive control because of its excellent safety profile and potent antioxidant properties. As shown in Figure 2b, PC‐Ca were much less likely to succumb to death after TAA exposure (*p*< 0.05), whereas PC and NAC did not significantly increase the survival ratio compared to that for PBS at different times. Serum ALT and AST levels were measured to assess the degree of damage to liver function. PLT levels were measured to evaluate coagulation function. The levels of ALT, AST, and PLT were abnormal in the ALF model mice, whereas PC‐Ca administration significantly improved serum ALT and AST, PLT levels compared with those in PC and NAC groups (Figure 2c–e). Compared with normal mice, in ALF model mice, the levels of inflammatory cytokines (IL‐6, TNF‐α) were higher, as determined by enzyme‐linked immunosorbent assay (ELISA). Similarly, compared with the PC and NAC groups, PC‐Ca notably reduced the levels of IL‐6, TNF‐α (Figure 2f,g). Moreover, the F4/80, IL‐6, and TNF‐α protein levels analyzed by immunohistochemistry (IHC) of liver tissues in the PC‐Ca group were lower than those in ALF, PC, and NAC groups (Figure 2h–k). Therefore, compared to NAC, PC‐Ca can significantly reduce inflammatory cell infiltration and inflammatory factor secretion.

**Figure 3:**
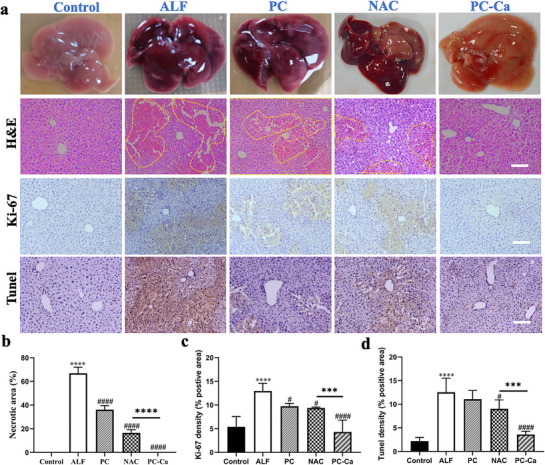
PC‐Ca alleviated the liver necrosis in TAA‐Induced ALF mice in vivo. a) Liver morphology in different groups. a,b) Representative H&E staining of liver tissues and quantification of hepatic necrotic area (the yellow areas represent necrotic area; Scale bar = 200 µm). (a,c) Representative Ki‐67 by immunohistochemistry (IHC) staining of liver tissues and quantification of Ki‐67 density of % positive area. (a,d) Representative Tunel staining of liver tissues and quantification of Tunel density of % positive area. ^*^ *p*< 0.05, ^**^ *p*< 0.01, ^***^ *p*< 0.001, ^****^ *p*< 0.0001 vs Control group. ^##^ *p*< 0.01, ^###^ *p*< 0.001, ^####^ *p*< 0.0001 vs ALF group).

Liver morphology revealed serious liver injury in the ALF group, PC group, and NAC group; however, there was no significant change in the control and PC‐Ca groups (**Figure** **3**a). TAA‐induced centrilobular necrosis and increased numbers of infiltrative inflammatory cells in the livers of the ALF group, as assessed by H&E staining. The area of hepatic necrosis and injury decreased in the PC and NAC groups, whereas the PC‐Ca group barely showed necrosis or inflammatory infiltration, and quantification was markedly attenuated (Figure 3a,b). In addition, TAA resulted in hepatocyte necrosis and induced hepatocyte proliferation. Ki‐67 and TUNEL staining are the most common indicators to evaluate proliferation and apoptosis. As shown in Figure 3a,c,d, the density of Ki‐67‐ and TUNEL‐positive areas was clearly elevated in the ALF and PC groups, but no such accumulation was observed in the PC‐Ca group. Collectively, these results show that TAA‐induced acute liver failure was more strongly attenuated in PC‐Ca than in PC and NAC groups

### 2.4 PC‐Ca Regulated KEAP1‐NRF2 Axis in the Defense Against Oxidative Assaults

**Figure 4:**
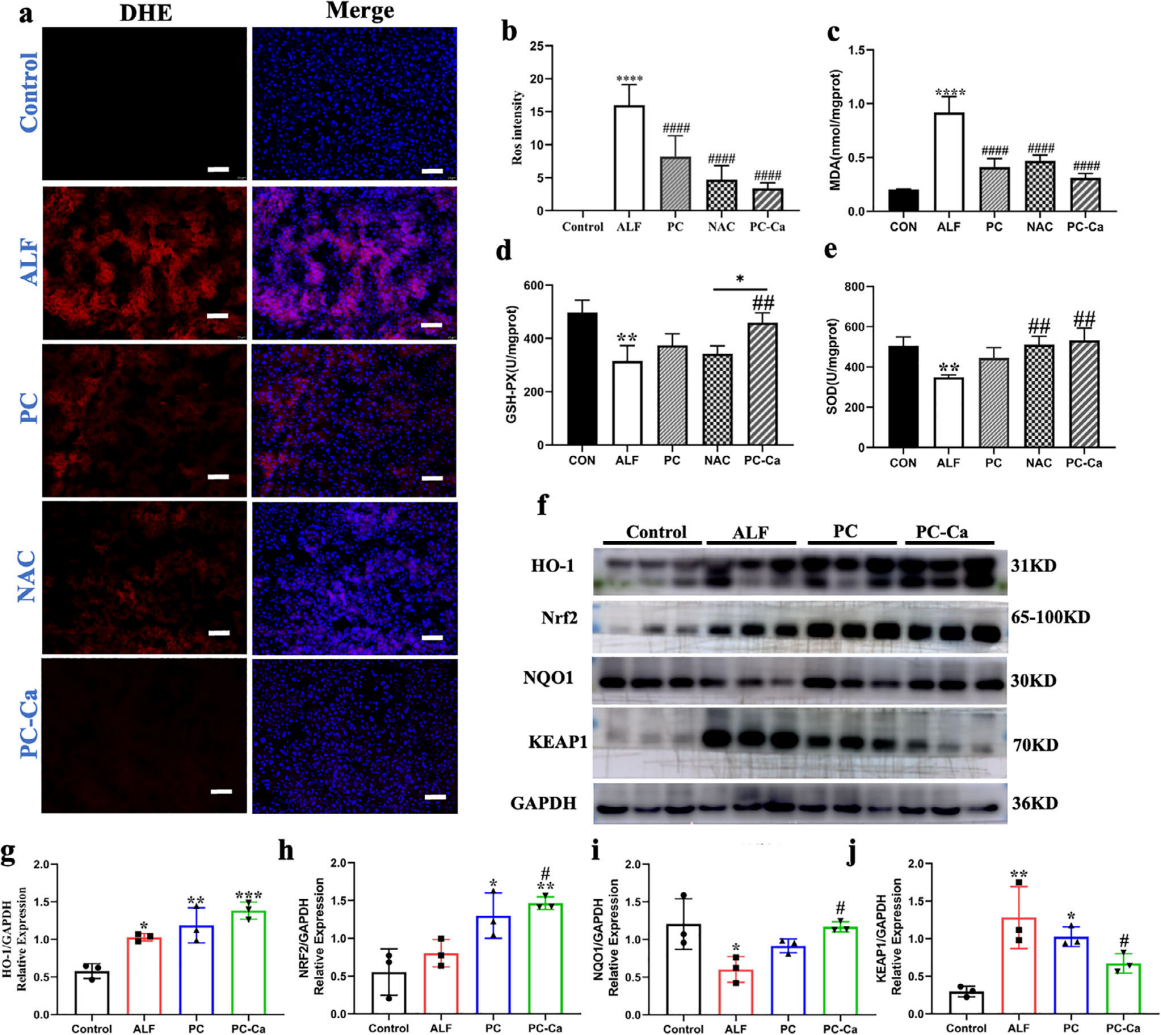
PC‐Ca regulated KEAP1‐NRF2 against oxidative assaults in TAA‐Induced ALF mice. a) Immunofluorescence analysis of ROS with DHE probe in frozen liver sections from mice. Red represents DHE (ROS), and Blue represents DAPI (cell nucleus). Scale bar = 100 µm. b) The fluorescent signal strength was quantified with ImageJ. At least 3 view fields under a 20x objective were quantified per section. c–e) Effects of PC‐Ca on oxidative stress in liver samples. (c) MDA level (d) GSH‐PX level. (e) Total SOD activity. f) Western blot analysis of the HO‐1, Nrf2, NQO1, and KEAP1 levels in each group. g–j) Densitometric analysis of the four proteins. ^*^ *p*< 0.05, ^**^ *p*< 0.01, ^***^ *p*< 0.001, ^****^ *p*< 0.0001 vs Control group, ^##^ *p*< 0.01, ^###^ *p*< 0.001, ^####^ *p*< 0.0001 vs ALF group).

TAA induces oxidative stress. The levels of ROS in frozen liver sections were analyzed by immunofluorescence using the ROS probe dihydroethidium (DHE) in the frozen liver sections. The accumulation of ROS in the ALF group was readily detectable. However, no such accumulation was observed in the PC‐Ca group, which was much lower than that in the PC and NAC groups according to the quantification (**Figure** **4**a,b). In addition, oxidative stress, which contributes to cell damage and death, was evaluated by measuring the related oxidative markers, including superoxide dismutase (SOD), malondialdehyde (MDA), and glutathione peroxidase (GSH‐Px). TAA induced high levels of MDA in the liver tissue compared to those in the control group. TAA reduced the levels of total SOD activity and GSH‐PX level in the liver tissue compared to those in the control group. However, PC, NAC, and PC‐Ca administration significantly restored the levels of SOD, MDA, and GSH‐PX after TAA exposure (Figure 4c–e). Next, we investigated the expression of the antioxidant proteins Nrf2, NQO1, and HO‐1, which are key transcription factors involved in defense against TAA‐induced liver injury. Compared to the control group, in the ALF group, the expression of Nrf2 and NQO1 was significantly lower. However, the expression of HO‐1 was pathologically higher in the PC and PC‐Ca groups compared to that in the ALF group (Figure 4f–i). PC‐Ca treatment significantly upregulated the expression of these two proteins after TAA stimulation compared to that in the PC group. Interestingly, the increased protein levels of KEAP1 were observed in the absence of the ALF group (Figure 4f,j). Similarly, a significant decrease in KEAP1 and an increase in NRF2 levels were observed in the PC‐Ca group (Figure 4j). Collectively, these results indicated that PC‐Ca regulates the KEAP1‐NRF2 axis against oxidative stress in TAA‐induced ALF mice.

### 2.5 RNA‐Seq Transcriptional Evidence Reveals that PC‐Ca can Modulate Necroptosis in TAA‐Induced ALF

**Figure 5:**
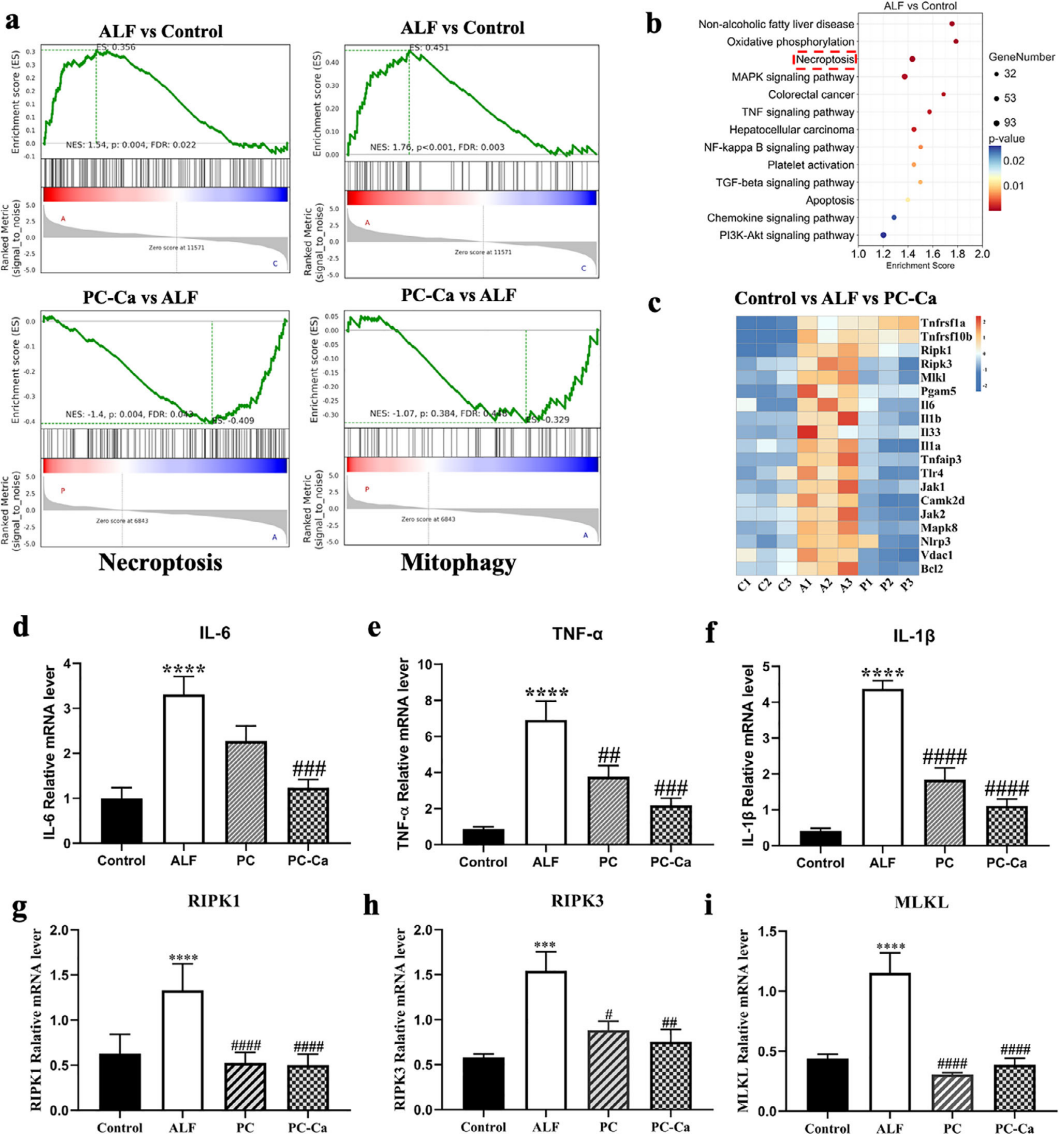
RNA‐seq transcriptional evidence reveals that PC‐Ca can modulate necroptosis in TAA‐induced ALF models. a) GSEA revealed that processes related to necroptosis and mitophagy genes were activated in the ALF group and repressed in the PC‐Ca group. b) Bubble diagram showed that the function of DEGs between the Control and ALF group was mainly enriched in necroptosis, oxidative phosphorylation, inflammation, metabolism, and cancer. c) Heatmap indicated that SDEGs are closely related to necroptosis, mitophagy, and inflammation (C1‐3 represented Control group 1–3, A1‐3 represented ALF group 1–3, P1‐3 represented PC‐Ca group1‐3). d–i) RT‐qPCR analysis of IL‐6, TNF‐α, IL‐1β, RIPK1, RIPK3, MLKL gene expression. (^****^ *p*< 0.0001 vs Control group, ^#^ *p*< 0.05, ^##^ *p*< 0.01, ^###^ *p*< 0.001, ^####^ *p*< 0.0001 vs ALF group).

To further confirm the above conclusions, we used high‐throughput RNA‐seq technology to assess the transcriptomes of livers from control (C1‐C3), TAA‐treated (A1‐A3), and TAA + PC‐Ca‐cotreated (P1‐P3) mice (n  =  3; data were analyzed on the free online platform of the Oebiotech Cloud Platform (https://www.oebiotech.com/). Correlations and principal component analyses (PCA) of the whole‐genome expression profiles showed that the control, ALF, and PC‐Ca groups clustered together and were separated from each other. Compared with the control group, the ALF group showed 2428 upregulated genes and 2392 downregulated genes. Compared to the ALF group, the PC‐Ca group showed 1597 upregulated and 2231 downregulated genes. Volcano and Venn plots showed 1753 differentially expressed genes between the three groups (Figure S8, Supporting Information). Gene set enrichment analysis (GSEA) revealed that, compared to the control group, the ALF group was significantly enriched and activated in the necroptosis and mitophagy pathways (NES values were 1.54 and 1.76, both positive, FDR < 0.05). Compared with the ALF group, the PC‐Ca group was also enriched in the necroptosis and mitophagy pathways but was inhibited (NES values were −1.4 and −1.07, both negative, FDR< 0.05). Therefore, these data indicate that cell necroptosis and mitophagy pathways were activated after TAA exposure, whereas they were inhibited by PC‐Ca treatment, consistent with the changes in cell function (**Figure** **5**a). As shown in Figure 5b, the bubble diagram of the KEGG pathway enrichment analysis revealed that the functions of differentially expressed genes (DEGs) between the control and ALF groups were mainly enriched in necroptosis, oxidative phosphorylation, inflammation, metabolism, and cancer. Importantly, cluster heatmap indicated that many more significantly differentially expressed genes (SDEGs) are closely related to necroptosis, mitophagy, and inflammation, such as RIPK1, RIPK3, MLKL, PGAM5, TNF‐α, IL‐6, IL‐1β (Figure 5c). To validate the expression results generated by RNA‐seq, the mRNA levels of necroptosis and inflammation SDEGs (IL‐6, TNF‐α, IL‐1β, RIPK1, RIPK3, MLKL) were determined by qRT‐PCR, and they were all downregulated in PC‐Ca‐cotreated mice compared to those in ALF mice (Figure 5d–i). RNA‐seq data demonstrated that PC‐Ca alleviated liver injury by regulating necroptosis and mitophagy.

### 2.6 PC‐Ca Protects Mitochondrial Function from Necroptosis

**Figure 6:**
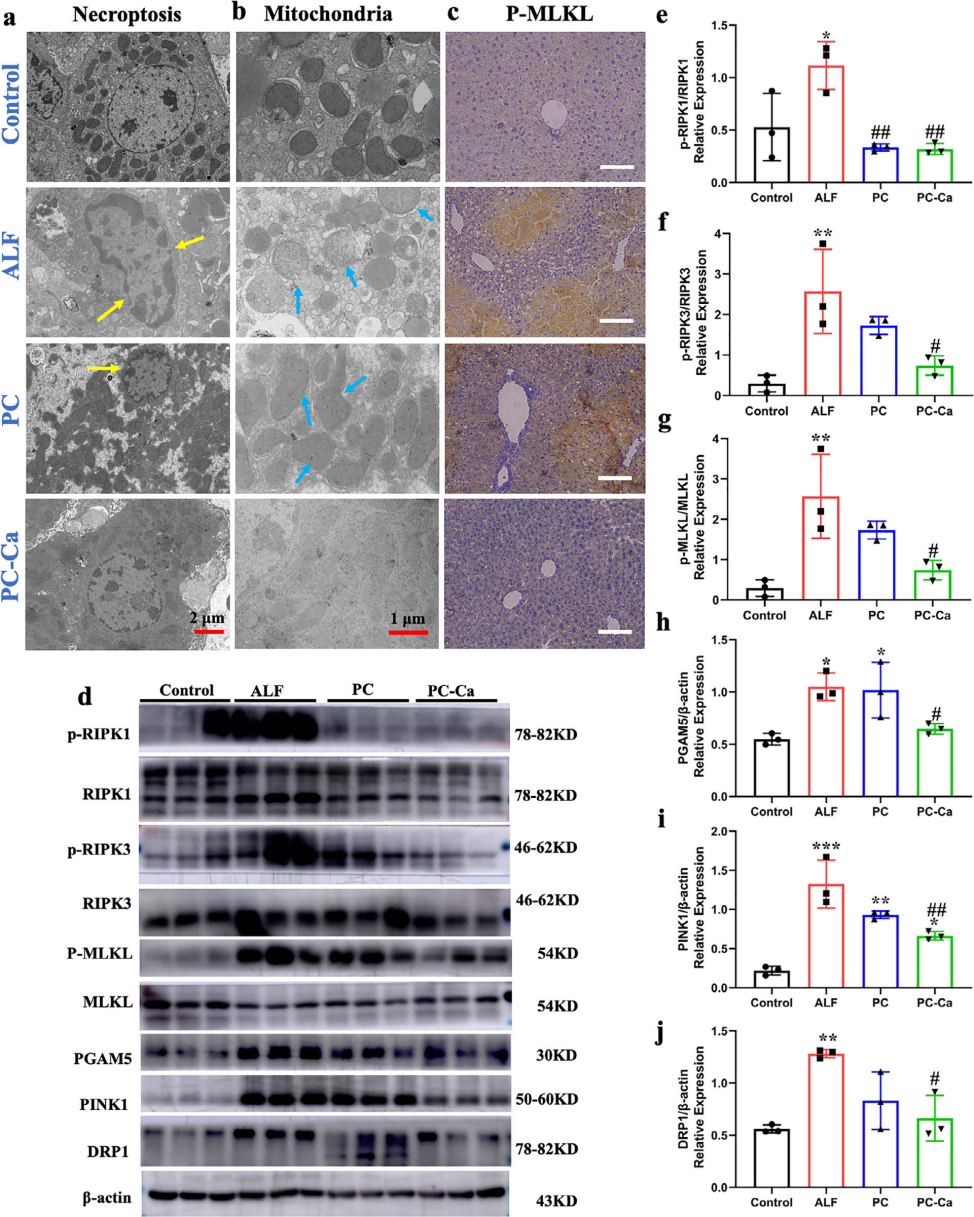
PC‐Ca protected mitochondrial function from necroptosis through PGAM5/DRP1/PINK1. a) TEM representative images about the morphology character of the cytomembrane and cell nucleus in liver tissues of each group (the yellow arrows represent membranolysis and karyolysis). Scale bar = 2 µm. b) TEM representative images about the mitochondrial morphology in liver tissues of each group (the blue arrows represent mitochondrial cristae ambiguity, tumidness, and disintegration). Scale bar = 1 µm. c) Representative p‐MLKL by immunohistochemistry (IHC) staining of liver tissues. Scale bar = 200 µm. d) Western blotting analysis of the RIPK1/RIPK3/MLKL and PGAM5/DRP1/PINK1 proteins isolated from the liver of each group of mice. Quantification of p‐RIPK1/ RIPK1 e), p‐RIPK3/ RIPK3 f), p‐MLKL/MLKL g), PGAM5/β‐actin h), DRP1/β‐actin j), PINK1/β‐actin i), relative protein levels of liver tissues. (^*^ *p*< 0.05, ^**^ *p*< 0.01, ^***^ *p*< 0.001, ^****^ *p*< 0.0001 vs Control group, ^##^ *p*< 0.01, ^###^ *p*< 0.001, ^####^ *p*< 0.0001 vs ALF group).

Necroptosis, a new form of programmed cell death, is typically initiated by the tumor necrosis factor receptor (TNFR). Necroptosis is characterized by membranolysis and karyolysis. Transmission electron microscopy (TEM) confirmed that membranolysis and karyolysis after TAA exposure were clearly detected in the ALF and PC groups compared to those in the control group, whereas the cytomembrane and cell nuclei were basically normal after PC‐Ca treatment (**Figure** **6**a, the yellow arrows). TEM also showed that the transformation of mitochondrial morphology, such as mitochondrial cristae ambiguity, tumidness, and disintegration, after TAA exposure was clearer in the ALF and PC groups than in the control group, whereas the cytomembrane and cell nucleus were nearly normal after PC‐Ca treatment (Figure 6b, the blue arrows). Mitochondrial dysfunction decreases ATP production. As shown in Figure S9 (Supporting Information), the ATP production in the TAA‐induced ALF group was markedly lower than that in the control group, whereas it was significantly higher in the PC‐Ca and NAC groups. The data showed that TAA‐induced ALF resulted in necroptosis, destruction of mitochondrial morphology, and a decrease in ATP production. Synthetic PC‐Ca inhibits necroptosis and protects mitochondrial morphology and function.

Mitochondrial homeostasis is closely associated with necroptosis. To further explore the mechanism of action of PC‐Ca in TAA‐induced ALF, the protein expression of RIPK1/RIPK3/MLKL and PGAM5/DRP1/PINK1 was detected by western blotting. As shown in Figure 6c–f, compared with the control group, in the ALF group, the relative protein levels and quantification of p‐RIPK1/RIPK1, p‐RIPK3/RIPK3, and p‐MLKL/MLKL were significantly higher. By contrast, these levels were significantly lower in the PC‐Ca‐treated group. However, the PC group did not exhibit significant necroptosis, suggesting that necroptosis was inhibited by PC‐Ca. Subsequently, mitochondrial function‐related targets such as PGAM5/DRP1/PINK1 were measured. The results showed that TAA exposure can interfere with the stability of mitochondrial function, while the protein levels of PGAM5/DRP1/PINK1 decreased in the absence of PC‐Ca treatment compared to those in the PC group (Figure 6c,g–i). Collectively, these results demonstrate that TAA exposure accelerates the formation of the RIPK1/RIPK3/MLKL necrosome complex to induce necroptosis, and that PC‐Ca may protect mitochondrial morphology and function through PGAM5/DRP1/PINK1, thereby increasing ATP production and ameliorating necroptosis.

### 2.7 Therapeutic Efficacy of TAA‐Induced Acute Liver Failure in Rabbits

**Figure 7:**
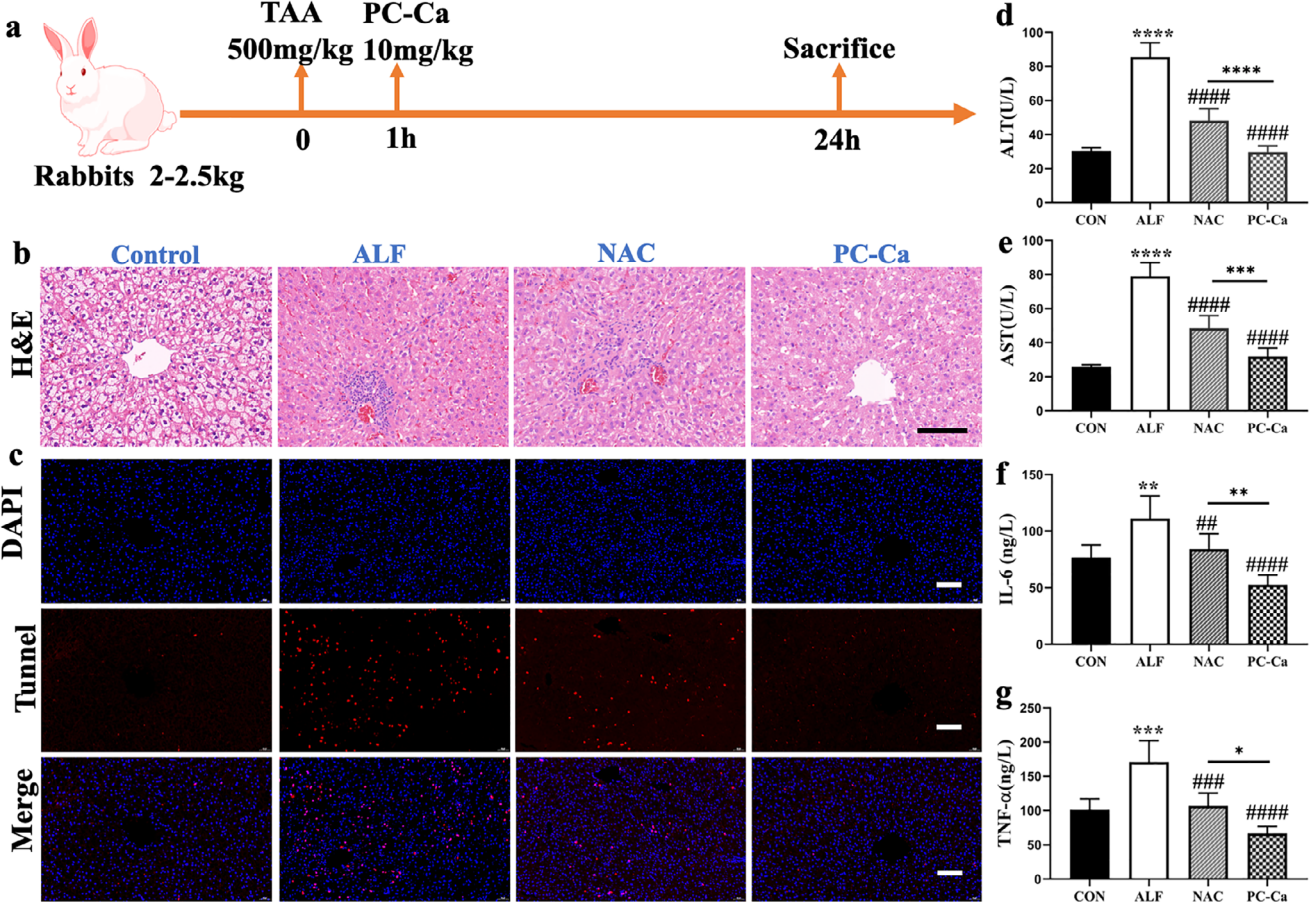
PC‐Ca alleviated the liver necrosis in TAA‐Induced ALF rabbits in vivo. a) The rabbits were injected intraperitoneally with 500 mg kg^−1^ TAA. After 1 h post‐modeling, the mice with TAA were injected intraperitoneally (i.p) with PC‐Ca (PC‐Ca, 10 mg kg^−1^). After 24 h post‐modeling, they were sacrificed for subsequent experiments. b) Representative H&E staining of liver tissues; Scale bar = 100 µm. c) Representative Tunel immunofluorescent staining of liver tissues in each group. d,e) The serum levels of ALT and AST at 24 h after TAA intoxication in rabbits. f,g) The levels of inflammatory cytokines (IL‐6, TNF‐α) by ELISA at 24 h after TAA intoxication in rabbits. ^**^ *p*< 0.01, ^***^ *p*< 0.001, ^****^ *p*< 0.0001 vs Control group, ^###^ *p*< 0.001, ^##^ *p*< 0.01, ^####^ *p*< 0.0001 vs ALF group).

To further verify the effects of PC‐Ca, we established a rabbit model of TAA‐induced ALF. As shown in **Figure** **7**a, rabbits were intraperitoneally injected with 500 mg kg^−1^ TAA. At 1 h post‐modeling, rabbits with TAA were injected intraperitoneally (i. p.) with PC‐Ca (10 mg kg^−1^). After 24 h, the rabbits were sacrificed for subsequent experiments. The area of hepatic necrosis and inflammatory cell infiltration increased in the ALF and NAC groups, whereas the PC‐Ca group barely showed necrosis or inflammatory infiltration on H&E staining (Figure 7b). In addition, TUNEL immunofluorescence (IF) staining was elevated in the ALF group, but no such accumulation was observed in the PC‐Ca group (Figure 7c). The levels of ALT and AST were both obviously abnormal in the ALF group, whereas PC‐Ca administration significantly improved serum ALT and AST levels compared to those in the NAC group (Figure 7d,e). Compared with normal mice, in ALF model mice, the levels of inflammatory cytokines (IL‐6, TNF‐α) were higher, as detected by ELISA. Similarly, compared to the NAC group, PC‐Ca reduced the levels of these cytokines (Figure 7f–g). These results also demonstrated that PC‐Ca effectively improved liver necrosis, liver function, and inflammatory infiltration in a TAA‐induced ALF rabbit model.

### 2.8 PC‐Ca Decreases ROS Formation of LO2 Cells In Vitro

**Figure 8:**
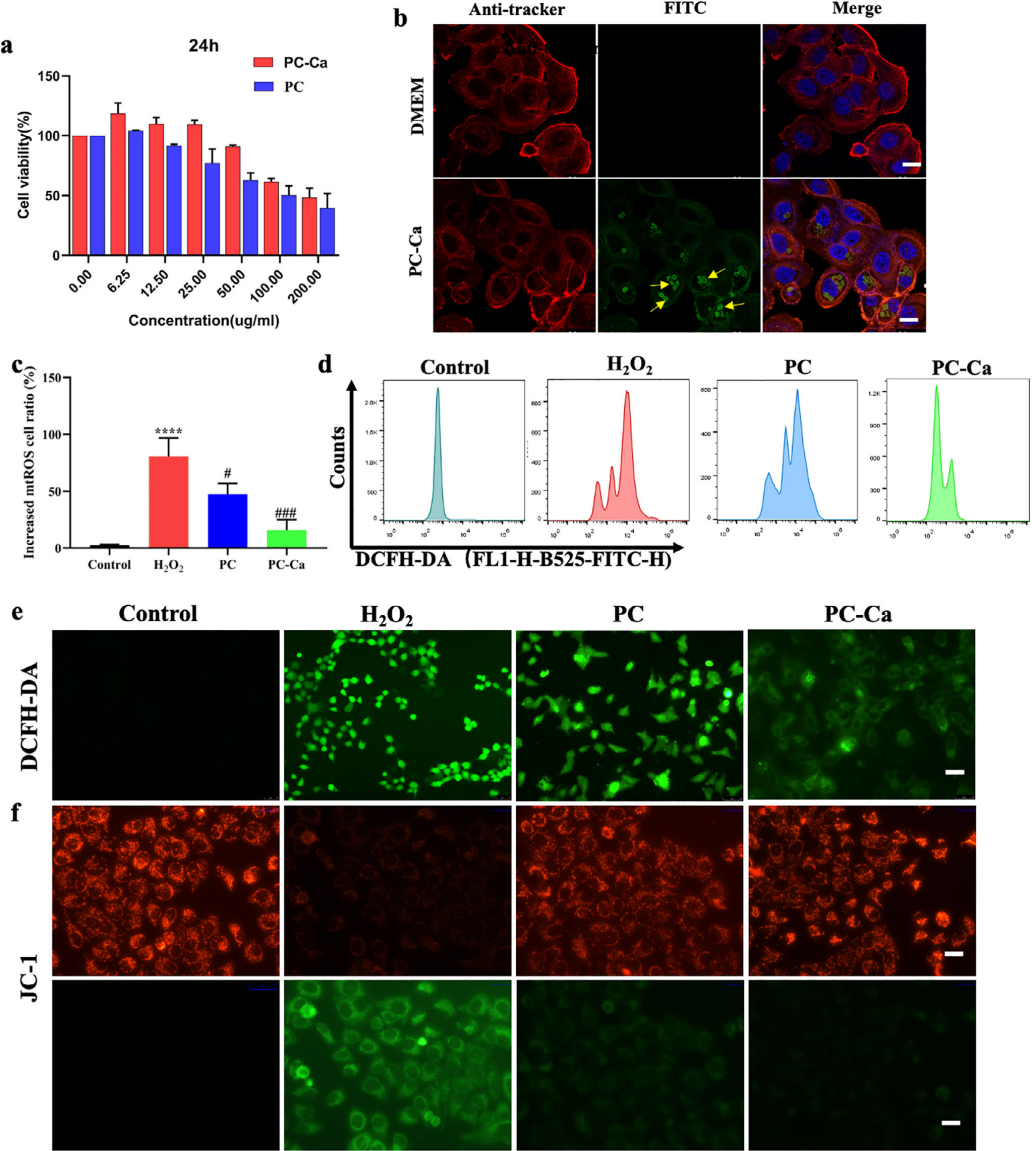
Inhibition of apoptosis and ROS Elimination of PC‐Ca in LO2 cells in vitro. a) Relative cell viability of LO2 cells in PC‐Ca and PC with different concentrations (0, 6.25, 12.5, 25, 50, 100, 200 µg mL^−1^) for 24 h by a CCK8 assay. b) The cellular uptake of PC‐Ca on LO2 cells by the confocal laser scanning microscopy (the yellow arrows represent PC‐Ca. The PC‐Ca has its own green fluorescence, with a wavelength similar to that of FITC). Scale bar = 20 µm. c,d) ROS levels in H_2_O_2_‐stimulated LO2 cells with different treatments by flow cytometry and quantification. e) Representative fluorescence images of H_2_O_2_‐stimulated LO2 cells with different treatments by DCFH probe staining. Scale bar = 50 µm. f) Representative fluorescence images of H_2_O_2_‐stimulated LO2 cells with different treatments by JC‐1 probe staining (green fluorescence represents monomer, red fluorescence represents aggregates). Scale bar = 50 µm. Data are expressed as means ± S.D. ^*^ *p*< 0.05, ^**^ *p*< 0.01, ^***^ *p*< 0.001, ^****^ *p*< 0.0001 vs Control group, ^##^ *p*< 0.01, ^###^ *p*< 0.001, ^####^ *p*< 0.0001 vs H_2_O_2_ group).

To evaluate the cytotoxicity in vitro, PC‐Ca and PC with different concentrations (0, 6.25, 12.5, 25, 50, 100, 200 µg mL^−1^) were incubated with normal human liver cell line (LO2 cells) for 24 h by a CCK‐8 (cell counting kit‐8 assay). There was no statistical difference in the relative cell viability of below 50 µg mL^−1^ concentration of PC‐Ca, whereas PC had obvious cytotoxicity when the concentration of PC was ≥50 µg mL^−1^ (**Figure** **8**a). Thus, PC‐Ca decreases the cytotoxicity of PC and has better biocompatibility with cells. The cellular uptake of PC‐Ca by LO2 cells was investigated using confocal laser scanning microscopy. The results suggested that PC‐Ca could be phagocytosed by LO2 cells (yellow arrows represent PC‐Ca, Figure 8b). To explore the potential cell‐protective mechanism of PC‐Ca, ROS levels in H_2_O_2_‐stimulated LO2 cells were investigated using flow cytometry and DCFH‐DA (2′,7′‐Dichlorodihydrofluorescein diacetate) probe staining. As shown in Figure 8c–f, exposure to H_2_O_2_ resulted in LO2 cells producing a significant amount of ROS and JC‐1 monomer/aggregate ratio, as determined by flow cytometry and fluorescence staining. Compared with the PC group, pretreatment with PC‐Ca significantly decreased ROS production and the JC‐1 monomer/aggregate ratio. Thus, the above results indicate that PC‐Ca inhibits necroptosis, eliminates ROS, and improves mitochondrial membrane potential to restore mitochondrial function.

## 3 Discussion

Procynaidin has long been recognized for their ability to mitigate oxidative stress and inflammatory responses. However, their therapeutic efficacy is typically less pronounced than that of NAC, which is consistent with the findings presented herein.^[^ ^32^, ^33^ ^]^ To overcome this limitation, we employed structural modifications to proanthocyanidins, leading to the development of PC‐Ca. Compared to polyphenol capsules based on metal coordination, these capsules are composed solely of PC, ensuring both biocompatibility and the absence of toxic or metal contaminants. Notably, these capsules maintained their structural integrity and functional activity during the test period (up to at least 1 month) in vitro, indicating their stability and sustained efficacy. Our results demonstrated that PC‐Ca exhibited significantly better free radical scavenging activity compared to that of free PC and VC, emphasizing its superior antioxidant properties. These findings suggested that PC‐Ca is a promising therapeutic candidate for the treatment of both acute and chronic liver diseases.

The pathogenesis of ALF is multifactorial and involves oxidative stress, sterile inflammation, and extensive hepatocyte death.^[^ ^34^, ^35^ ^]^ Current therapeutic options for ALF are limited and typically comprise supportive care, NAC administration for acetaminophen (APAP) overdose, corticosteroids, artificial liver support devices, and liver transplantation. NAC has been shown to restore glutathione levels and aid detoxification in the early stages of ALF and is considered a safe and cost‐effective treatment when administered within 8 h of APAP overdose. However, its efficacy declines sharply beyond this window, with mortality rates increasing by 40%.^[^ ^36^ ^]^ In advanced ALF cases, liver transplantation remains the definitive treatment, although it is hindered by limited donor availability and high costs. In this context, our study found that PC‐Ca preferentially accumulates in the liver tissue following injury, passively targeting the organ. Notably, PC‐Ca treatment significantly alleviated hepatocyte necrosis and proliferation and improved survival rates as well as liver function in TAA‐induced ALF animal models. In addition, PC‐Ca reduced inflammatory cell infiltration and cytokine secretion. This is consistent with the previous research results of our research group.^[^ ^37^, ^38^ ^]^ Importantly, the superior therapeutic efficacy of PC‐Ca was demonstrated in two animal models, suggesting its potential as an alternative to NAC in the management of human ALF.

Mitochondria play a central role in cellular energy metabolism, and mitochondrial dysfunction is a hallmark of numerous diseases, including ALF.^[^ ^39^ ^]^ Hepatic injury disrupts mitochondrial function and triggers a cascade of oxidative stress, energy depletion, steatosis, and necrosis. Oxidative stress is a critical factor in ALF pathogenesis, and the KEAP1‐NRF2 pathway has emerged as a key regulator of the cellular defense against oxidative damage.^[^ ^40^ ^]^ In the TAA‐induced ALF model, mitochondrial dysfunction and inflammation resulted in elevated ROS levels, activating the antioxidant response via the upregulation of HO‐1 and NQO1 through the KEAP1‐NRF2 signaling pathway. PC‐Ca effectively mitigated ROS accumulation and enhanced the expression of antioxidant enzymes, thereby protecting hepatocytes by preserving mitochondrial integrity and function. This protective mechanism not only highlights the therapeutic potential of PC‐Ca but also suggests its broader applicability in diseases characterized by oxidative stress and mitochondrial dysfunction. Targeting of this pathway may lead to the development of effective therapeutic strategies for ALF and related disorders.

Necroptosis, a form of programmed cell death, contributes significantly to liver injury and inflammation. ROS are the key mediators of necroptosis and are involved in the formation of the necrosome complex.^[^ ^41^ ^]^ RNA‐seq revealed that TAA‐induced ALF triggered necroptosis, a process markedly inhibited by PC‐Ca. Our study employed bioinformatics approaches to demonstrate that TAA exposure induced the formation of the RIPK1/RIPK3/MLKL necrosome complex, which was significantly suppressed by PC‐Ca treatment. Additionally, PC‐Ca reduced the expression of DRP1 and PINK1, which are key proteins involved in mitochondrial fission, by targeting PGAM5. This suppression helps preserve mitochondrial morphology and function and mitigates hepatocyte necrosis in ALF. The preservation of mitochondrial integrity is crucial for ensuring comprehensive hepatocyte protection and facilitating the restoration of hepatic function. Furthermore, PC‐Ca attenuated the release of proinflammatory cytokines, providing dual anti‐necroptotic and anti‐inflammatory effects. This dual action enhances the therapeutic potential of PC‐Ca, making it a promising candidate for the treatment of ALF and suggesting advantages over current treatment options.

## 4 Conclusion

We developed PC‐Ca, a mitochondria‐targeted therapeutic agent with robust anti‐inflammatory and antioxidant properties, that effectively alleviates TAA‐induced ALF. Mechanistically, PC‐Ca preserved mitochondrial integrity and inhibited necrotic pathways by selectively modulating the PGAM5/DRP1/PINK1 signaling axis, restoring mitochondrial dynamics, and enhancing mitophagy. These actions significantly improved the survival rates and hepatic function recovery in ALF models. Our findings emphasize the translational potential of mitochondria‐targeted therapies in ALF, positioning PC‐Ca as a promising alternative to NAC, which is the current clinical standard with limited efficacy in advanced ALF. The superior therapeutic outcomes of PC‐Ca, particularly in mitigating mitochondrial ROS overproduction and interrupting the self‐reinforcing cycle of mitochondrial dysfunction, underscore its potential to meet critical and unmet clinical needs. However, the study's limitations, including small sample size, reliance on animal models, and absence of clinical validation, underscore the need for further investigation to fully establish the clinical efficacy, safety, and translational potential of PC‐Ca in human therapy.

## 5 Experimental Section

### Materials

The LO2 cell line was obtained from iCell (Shanghai, China). DMEM, fetal bovine serum (FBS), penicillin‐streptomycin (Pen‐Strep), and trypsin‐EDTA solution were supplied by Gibco (USA). PBS was purchased from SolarBio (China). All chemicals, including proanthocyanidins (PC), sodium carbonate (Na_2_CO_3_), calcium chloride (CaCl_2_), hydrochloric acid (HCl), sodium hydroxide (NaOH), and thioacetamide (TAA), were provided by MACKLIN (Shanghai, China). N‐acetylcysteine (NAC) was purchased from Sigma (USA). Cell counting kit‐8 (CCK‐8) was purchased from GLPBIO (USA). Reactive Oxygen Species (ROS), adenosine triphosphate (ATP), Actin‐Tracker Green‐594, Hoechst 33342, Colorimetric TUNEL Apoptosis Assay Kit, antifade mounting medium with 2‐(4‐Amidinophenyl)‐6‐indolecarbamidine dihydrochloride (DAPI), and bicinchoninic acid (BCA) assay kit were obtained from Beyotime (Shanghai, China). ELISA for IL‐6 and TNF‐α was obtained from Boyun Biotech (Shanghai, China). Alanine aminotransferase (ALT), aspartate aminotransferase (AST), Total Superoxide Dismutase (T‐SOD), Glutathione Peroxidase (GSH‐Px), and MDA assay kits were purchased from Jiancheng (Nanjing, China). Anti‐Ki‐67 antibody was purchased from HUABIO (Hangzhou, China). Anti‐Keap1, anti‐NQO1, anti‐PGAM5, anti‐PINK1, and anti‐DRP1 antibodies were purchased from Proteintech (Wuhan, China). Anti‐F4/80, Anti‐IL‐6, anti‐TNF‐α, anti‐HO‐1, and anti‐NRF2 antibodies were obtained from Abcam (UK). Anti‐MLKL, anti‐p‐MLKL, anti‐RIPK1, anti‐p‐RIPK1, anti‐RIPK3, and anti‐p‐RIPK3 antibodies were purchased from Affinity Biosciences (Jiangsu, China).

### Synthesis of PC‐Ca

PC‐Ca was synthesized according to the method reported in the previous studies.^[^ ^42^ ^]^ Briefly, PC (800 mg) was dissolved in deionized water (10 mL), and 2 m sodium hydroxide was added to adjust the pH to 9 for the dissolution of polycarbonate. The preparation method for PC@CaCO3 and capsules was as follows: initially, a CaCl_2_ solution (3 mL, 0.33 m) was poured into a conical flask containing Na_2_CO_3_ (2 mL, 0.5 m) and PC (1 mL) at a set concentration (SoCon, 5, 10, 20, 40, 60, and 80 mg mL^−1^). After stirring at 1200 rpm for 40 s, the mixture was left undisturbed for 15 min, during which time the microparticles were formed. The microparticles were collected at 1000 revolutions per minute for 3 min and washed with deionized water. The centrifugation and washing processes were repeated three times. After the supernatant was removed, polycarbonate capsules were prepared by adding 50 mM EDTA and shaking for 4 h or by adding 1 m hydrochloric acid and shaking for 10 s. Finally, polycarbonate capsules were obtained by centrifugation at 3000 rpm for 5 min and washed with deionized water.

### Character of PC‐Ca

The prepared polycarbonate capsules were used in the subsequent experiments. The morphology of the microcapsules was observed using a scanning electron microscope (SEM, SU8010 HITACHI). A confocal laser scanning microscope (CLSM) mounted on a Nikon A1 device was used to obtain micrographs of the capsules and cells. Optical microscopy images were captured using a NIKON Ni‐U microscope, and bright‐field images were obtained using a 10x objective lens. Fourier‐transform infrared (FTIR) spectroscopy using a Bruker TENSOR II spectrometer was employed to measure the infrared spectra of the capsules and their constituent materials. X‐ray Photoelectron Spectroscopy (XPS) was acquired using a Thermo‐Electron ESCALAB 250 spectrometer.

### Free Radical Scavenging Assays of PC‐Ca

The total antioxidant capacity of PC‐Ca was assessed using a Ferric Reducing Antioxidant Power (FRAP) assay kit, following the manufacturer's instructions. The absorbance was measured at 593 nm using a Lambda 25 spectrophotometer (PerkinElmer). The DPPH (2,2‐diphenyl‐1‐picrylhydrazyl) radical scavenging activity of the vitamin C (VC), PC, and PC‐Ca solutions was determined using a DPPH radical scavenging assay kit according to the manufacturer's instructions. The absorbance at 515 nm was measured using a Lambda 25 spectrophotometer (PerkinElmer). The DPPH radical scavenging rates of the samples were calculated using the following formula:

(1) $$\begin{array}{cc} DPPHinhibitionrate\left(\%\right) & =\left\{Absblank-\frac{\left(Abscontrol-Abssample\right)}{Absblank}\right\}\;\;\\ & \;\times 100\% \end{array}$$

The H_2_O_2_ scavenging activities of the VC, PC, and PC‐Ca solutions were determined using the FOX (Fenton reaction‐based assay) method. The absorbance was measured at 560 nm using a UV–vis spectrophotometer. Based on the standard curve of H_2_O_2_, the H_2_O_2_ scavenging activity of the VC solution, PC solution, and PC‐Ca was determined.

(2) $$\begin{array}{cc} ABTSinhibitionrate\left(\%\right) & =\left\{Absblank-\frac{\left(Abscontrol-Abssample\right)}{Absblank}\right\}\;\;\\ & \;\times 100\% \end{array}$$

### In Vivo Biodistribution

When preparing PC‐Ca, Escherichia coli transfected with the mCherry protein was also added to the PC solution and CaCO_3_ solution to mark PC‐Ca, and mCherry‐PC‐Ca was obtained. To analyze the biodistribution of mCherry‐PC‐Ca in vivo, mice were deprived of food overnight, and thoracic and abdominal hair were removed before imaging. The next day, mice were intraperitoneally injected with 10 mg kg^−1^ mCherry‐PC‐Ca according to the control and TAA groups (NS and 300 mg kg^−1^ TAA, respectively), anesthetized using isoflurane, and photographed using an IVIS Lumina II in vivo imaging system at 1, 3, 6, and 24 h post‐injection. For ex vivo fluorescence imaging, the mice were sacrificed to collect their main organs (heart, liver, spleen, lungs, and kidneys) for tissue distribution analysis using the IVIS Lumina II in vivo imaging system 24 h post‐injection. And the liver tissues were extracted, then deparaffinized, hydrated, and cut into 5 µm‐thick sections. Liver tissues were stained with DAPI for confocal laser scanning microscopy (Leica Stellaris 5; Wetzlar, Germany) to detect the biodistribution of mCherry‐PC‐Ca in the liver.

### Animal Experiments

### Animal Experiments—Animal Models

All animal experiments were conducted in accordance with the guidelines approved by the Ethics Committee of the Wenzhou Institute, University of Chinese Academy of Sciences (WIUCAS24022202) and Laboratory Animal Ethics Committee of the First Affiliated Hospital of Wenzhou Medical University (WYYY‐AEC‐2025‐192). All the rabbits were individually housed in steel cages at the Laboratory Animal Center under standard feeding conditions throughout the experimental period. All the mice were housed under specific pathogen‐free (SPF) conditions throughout the experimental period. TAA‐induced acute liver failure models were established according to standard protocols. Briefly, mice and rabbits were injected intraperitoneally (i.p.) with saline as the control group. The rabbits were intraperitoneally injected with 500 mg kg^−1^ TAA and randomly divided into four groups (four rabbits per group). At 1 h post‐modeling, TAA‐treated rabbits were injected intraperitoneally (i. p.) with PBS, NAC (100 mg kg^−1^), or PC‐Ca (10 mg kg^−1^). Next, the mice were intraperitoneally injected with 300 mg kg^−1^ TAA and randomly divided into four groups (six mice per group). One hour post‐modeling, TAA‐treated mice were injected intraperitoneally (i. p.) with PBS, PC (100 mg kg^−1^), NAC (100 mg kg^−1^), or PC‐Ca (100 mg kg^−1^). All the mice and rabbits were allowed access to water and food ad libitum. After 24 h post‐modeling, the mice were sacrificed to collect serum and heart, liver, spleen, lung, and kidney tissues for subsequent experiments.

### Animal Experiments—Survival Curves of TAA‐Induced Acute Liver Failure in Mice

Mice were intraperitoneally injected with 300 mg kg^−1^ TAA. 1 h later, the mice were treated with PBS (i.p.), PC (100 mg kg^−1^, i.p.), NAC (100 mg kg^−1^, i.p.), or PC‐Ca (100 mg kg^−1^, i.p.) (n = 10). Subsequently, the mouse survival was monitored regularly.

### Animal Experiments—Blood Biochemical Assay and Blood Cell Counts

Blood samples were analyzed to test for platelets (PLT) using an Automated Hematology analyzer (BC‐5000 Vet). Blood samples were centrifuged to obtain serum, and the levels of aspartate aminotransferase (AST) and alanine aminotransferase (ALT) were measured to assess hepatic function according to the manufacturer's protocols. Additionally, ELISA was performed to detect the serum levels of TNF‐α and IL‐6 to evaluate the inflammation level following the manufacturer's instructions.

### Animal Experiments—Pathological Evaluation

Heart, liver, spleen, lung, and kidney tissues from the mice and the rabbits were extracted, then deparaffinized, hydrated, and cut into 5 µm‐thick sections. According to standard Protocols, H&E, Tunel immunofluorescent (IF) staining, immunohistochemistry (IHC) staining with Ki‐67 (1:5000) antibodies, F4/80 (1:500) antibodies, IL‐6 (1:100) antibodies, and TNF‐α (1:400) antibodies were performed, respectively. Representative images were captured using an optical microscope and quantified using ImageJ software.

### Animal Experiments—ROS Production Measurement In Vivo

To assess ROS production in liver tissues, fresh liver tissues were embedded in Optimal Cutting Temperature (OCT) and immediately frozen. Then samples were sliced into 6 µm‐thick sections and put on glass slides. After gently washing with PBS three times, DHE solution (5 µmol L^−1^) was applied to the sections for 15 min at 37 °C in the dark. After washing with PBS, the slides were mounted using DAPI and coverslips. Images were captured using a fluorescence microscope (Leica, Germany).

### Animal Experiments—Adenosine Triphosphate (ATP) Assay Kit and Enzyme Activity Assay

Liver tissue was accurately weighed, and pre‐cooled normal saline (weight (g): volume (mL) = 1:10) was added. The tissue sample was ground and centrifuged, and the supernatant was collected. Adenosine triphosphate (ATP) level was measured using an ATP Assay Kit (Beyotime) according to the manufacturer's protocols. For enzyme activity analysis, the activity of SOD, GSH‐PX, and MDA was analyzed using the Total Superoxide Dismutase (T‐SOD) assay kit, the Glutathione Peroxidase (GSH‐PX) assay kit, and the MDA assay kit according to the protocol.

### Animal Experiments—Western Blotting

Briefly, liver tissue samples were lysed in RIPA buffer containing phenylmethanesulfonyl fluoride (PMSF) and phosphatase inhibitors to obtain total proteins, and the lysates were centrifuged at high speed to remove insoluble debris. Protein concentration in the lysates was determined using a bicinchoninic acid (BCA) assay kit. Then, the lysates were separated by SDS‐PAGE and transferred onto PVDF membranes. After blocking for 1 h at room temperature, the membranes were incubated with the corresponding primary antibodies overnight at 4 °C. After incubation with the corresponding horseradish peroxidase (HRP)‐conjugated secondary antibody (1:5000), the membranes were imaged using a chemiluminescence system (GE AI600, USA). The levels of related proteins, including Keap1, NQO1, HO‐1, NRF2, MLKL, p‐MLKL, RIPK1, p‐RIPK1, RIPK3, p‐RIPK3, PGAM5, PINK1, and DRP1, in liver tissues from different groups were detected by western blotting. The protein levels were normalized to β‐actin and GAPDH as reference proteins. Quantification of the blots was performed using the ImageJ software.

### Animal Experiments—RT‐PCR

Total RNAs were isolated from the liver using an RNA Simple Total RNA Kit. The RNA concentration was determined using a Nanodrop 2000 (Thermo Scientific). RT‐PCR was performed using a commercial two‐step kit and analyzed using the 2× Taq Pro Universal SYBR Green Master Mix. The mRNA levels of IL‐6, TNF‐α, MLKL, RIPK1, and RIPK3 were examined by qRT‐PCR using a 7500 Fast Real‐Time PCR System (Applied Biosystems). GAPDH was used as an endogenous control for normalization. Table S1 (Supporting Information) lists the primer sequences used in this study.

### Animal Experiments—RNA‑seq

Total RNA was isolated from the liver tissues using TRIzol reagent (Invitrogen, CA, USA). Libraries were constructed using the VAHTS Universal V6 RNA‐seq Library Prep Kit according to the manufacturer's instructions. Transcriptome sequencing was performed on an Illumina platform (Illumina NovaSeq 6000, China), and 150 bp paired‐end reads were generated. Raw sequencing reads for each sample were trimmed to remove low‐quality reads and adapters using Fast software (version 0.22.0). Clean reads were mapped to the reference genome using HISAT2. The read counts of each gene were obtained using HTSeq‐count. DESeq2 was used to normalize gene expression and detect differentially expressed genes (*p*< 0.05, fold change >2, fold change< 0.5). Gene set enrichment analysis (GSEA software) was used for functional enrichment analysis of the Gene Ontology Biology process for DEGs. Transcriptome sequencing and analysis were performed by OE Biotech Co., Ltd. (Shanghai, China).

### Animal Experiments—TEM of Liver Tissues

Liver tissue (1 mm^3^) was fixed overnight with 2.5% glutaraldehyde solution at 4 °C for 24 h. The fixing solution was poured away, and the sample was rinsed three times with PBS 0.1 m and pH 7.4 for 15 min each time. The sample was fixed with 1% osmic acid solution for 1–2 h. The osmic acid waste solution was carefully removed, and the sample was rinsed with phosphoric acid buffer three times for 15 min each. The samples were dehydrated with an ethanol solution of gradient concentration (30%, 50%, 70%, 80%, 90%, and 95%) for 10 min, followed by treatment with 100% ethanol for 20 min. Finally, the mixtures were treated with acetone for 20 min. The sample was treated with a mixture of embedding agent and acetone (V/V = 1/1) for 1 h, followed by treatment with a mixture of embedding agent and acetone (V/V = 3/1) for 3 h. The sample was treated with pure embedding agent overnight; the permeated sample was then embedded and heated at 60 °C overnight. The samples were sliced using an ultrathin microtome (LEICA UC7, Germany), and slices of 70–90 nm thickness were obtained. The slices were stained with a 50% ethanol‐saturated solution of uranyl acetate for 8–15 min and lead citrate solution for 8–10 min, respectively, and then observed under a transmission electron microscope (Hitachi H‐7650).

### Cell Culture Experiments

### Cell Culture Experiments—Cell Biocompatibility

LO2 cells were seeded in 96‐well plates at a density of 1 × 10^4^ cells well^−1^ in DMEM (containing 10% FBS) in 5% CO_2_ at 37 °C. After culturing for 12 h, removing the culture medium and washing with PBS, different concentrations were added (0, 6.25, 12.5, 25, 50, 100, 200 µg mL^−1^) of PC‐Ca and PC solution with 10% DMEM for 24 h. Cell biocompatibility was evaluated using a CCK‐8 assay.

### Cell Culture Experiments—Cell Uptake Efficiency

LO2 cells were seeded in confocal dishes at a density of 2 × 10^5^ cells well^−1^. After 12 h of incubation, PC‐Ca (5 µg mL^−1^) was added and incubated with LO2 cells for another 24 h. LO2 cells were then fixed, cell nuclei were stained with DAPI, and the cell cytoskeleton was stained with an anti‐tracker. PC‐Ca exhibited green fluorescence (similar to that of the FITC channel). Images were obtained using a confocal laser‐scanning microscope (Leica Stellaris 5, Germany).

### Cell Culture Experiments—ROS Production Measurement

Briefly, the cells were retreated with PC or PC‐Ca (12.5 µg mL^−1^) overnight and then incubated with H_2_O_2_ (1000 µM) for 2 h to induce oxidative stress. Intracellular ROS production was monitored using the fluorescent probe DCFH. The cells were stained with DHE according to the manufacturer's instructions. The cells were then washed with PBS and analyzed using flow cytometry (CytoFLEX, USA) or a fluorescent microscope (Leica, Germany).

### Cell Culture Experiments—Mitochondrial Membrane Potential Measurement

LO2 cells were pretreated with the PC or PC‐Ca (12.5 µg mL^−1^) overnight. The cells were incubated with H_2_O_2_ (1000 µM) for another 2 h and then stained with JC‐1 dye for 20 min at 37 °C, washed third with JC‐1 buffer. The fluorescence of JC‐1 was analyzed using a fluorescence microscope (Leica, Germany).

### Statistical Analysis

Gray scale analysis of protein bands, ROS fluorescence intensity, and areas of necrosis in liver tissues were analyzed using ImageJ software (National Institutes of Health, Maryland, USA). Survival curves were generated using Kaplan–Meier plots and analyzed using log‐rank analysis. Experimental data were expressed as the Mean ± SD, and accessed by using GraphPad Prism 9.0 (GraphPad, USA). Significant differences between the groups were analyzed using ANOVA. Statistical significance was determined by ^*^ *p*< 0.05, and ^**^ *p*< 0.01.

## Conflict of Interest

The authors declare no conflict of interest.

## Author Contributions

Q.S. and M.W. contributed equally to this work. Q.S. and X.Z. conceived the conceptualization and designed the experiment. M.W., J.Z., C.C., Z. H., J.P. and D.Y. carried out the experiments and analyzed the data. M.W. and Q.S. wrote the paper. D.Y., X.Z., and Z.W. edited the manuscript. All authors have read and approved the publication of the manuscript.

## Supporting information



Supporting Information

## Data Availability

The data that support the findings of this study are available from the corresponding author upon reasonable request.
