# Supplementary material for: Procyanidin Capsules Combat ALF by Restoring Mitochondrial Homeostasis and Inhibiting Necroptosis via the PGAM5/DRP1/PINK1 Pathway
Source: Adv Sci (Weinh). 2025 Dec 3;13(7):e08742. doi: 10.1002/advs.202508742 (PMC12866713; doi:10.1002/advs.202508742)
Supplement: Supplementary file 1 — Supporting Information [file ADVS-13-e08742-s001.docx]

**Supplementary Material**

**Procyanidin Capsules Combat ALF by Restoring Mitochondrial Homeostasis and Inhibiting Necroptosis via the PGAM5/DRP1/PINK1 Pathway**

Qing Shi^a,b, #^, Minmin Wu^b, #^, Jinwei Zhong^a^, Chao Chen^a^, Zhuang Huang^c^, Jingxuan Peng ^c^, Dong Yang ^d *^, Xingjie Zan^d *^, and Zhengfei Wang^e *^

^a^ Department of Gastroenterology, The First Affiliated Hospital of Wenzhou Medical University, Wenzhou, 325035, China

^b^ Zhejiang Key Laboratory of Intelligent Cancer Biomarker Discovery and Translation, The First Affiliated Hospital of Wenzhou Medical University, Wenzhou, 325035, China

^c^ Wenzhou Medical University, Wenzhou, 325035, China

^d^ Wenzhou Institute, Wenzhou Key Laboratory of Perioperative Medicine, University of Chinese Academy of Sciences, Wenzhou, 325001, China

^e^ The Quzhou Affiliated Hospital of Wenzhou Medical University, Quzhou People's Hospital, Quzhou Zhejiang Province, China. 324000

^#^ Equal contribution author.

^*^ Corresponding author.

Email: [602024240153@smail.nju.edu.cn](mailto:602024240153@smail.nju.edu.cn) (D. Yang); zanxj@ucas.ac.cn (X. Zan); wangzhengfei1405@wmu.edu.cn (Z. Wang);


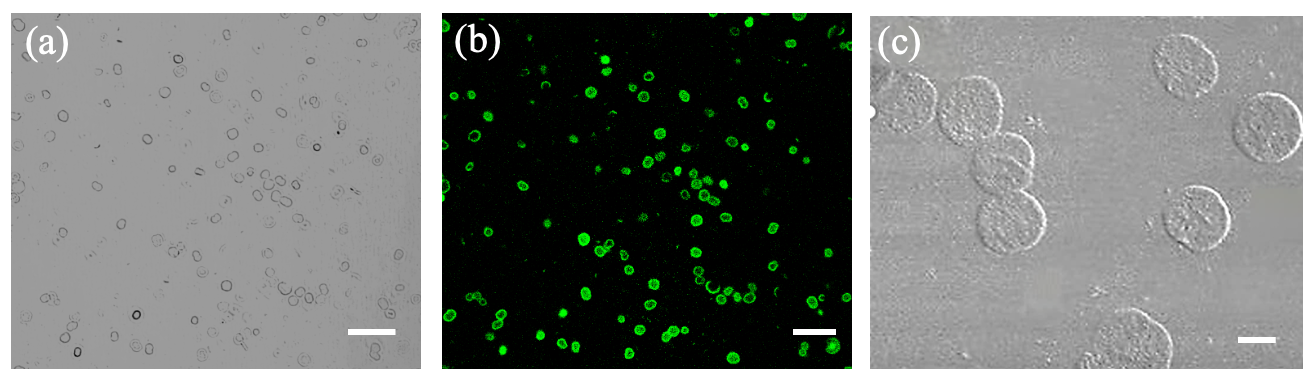


**Figure S1.** The Preparation and Characterization of PC/Ca Capsules. (a) OM, (b) CLSM, (c) SEM of PC-Ca capsules. The scare bars in (a) and (b) are 20 μm. The scare bars in (c) is 3 μm.


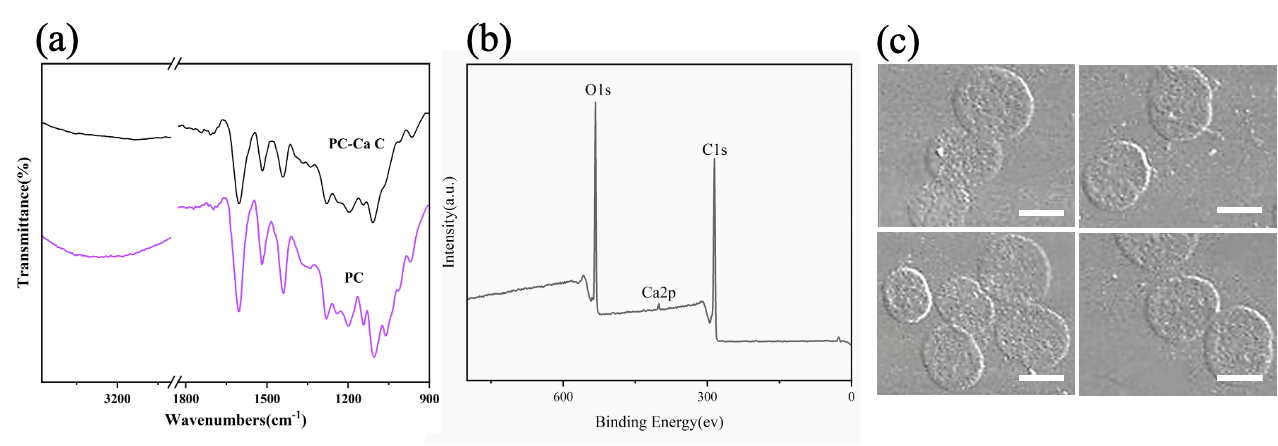


**Figure S2.** (a) Fourier transform infrared (FTIR) spectra of PC and PC capsules. (b) XPS spectra of PC capsules. (c) Stability of capsules in solution of (i) pure water, (ii) PBS solution, (iii) 0.9% NaCl and (iv) DMEM. The scare bars in (c) is 3 μm.


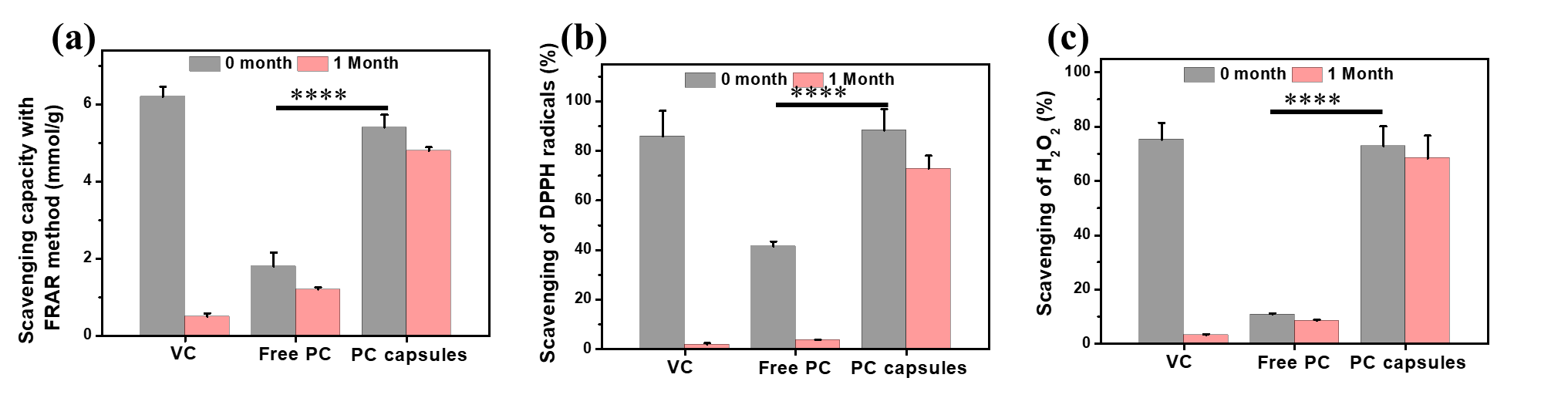


**Figure S3.** (a) Total antioxidant ability, (b) DPPH radical scavenging ability and (c) ABTS radical scavenging ability of Vc, PC and PC-Ca capsules. The data on the left of Vc, PC and PC capsule in (a), (b) and (c) represent short-term antioxidant effects, and the right represent the long-term scavenging effects. (P values: ****P < 0.0001, all the values are expressed as mean ± SD, n = 3).


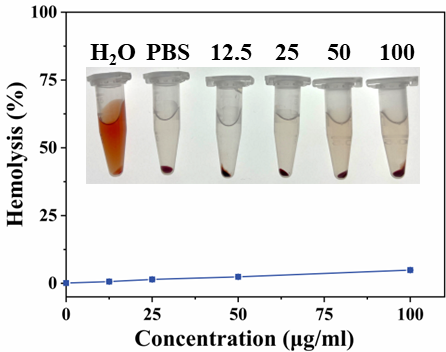


**Figure S4.** The hemolytic effect of PC capsules, with PBS as a negative control and ddH_2_O as a positive control. The number of PC capsules ranged from 12.5 to 100ug/ml.


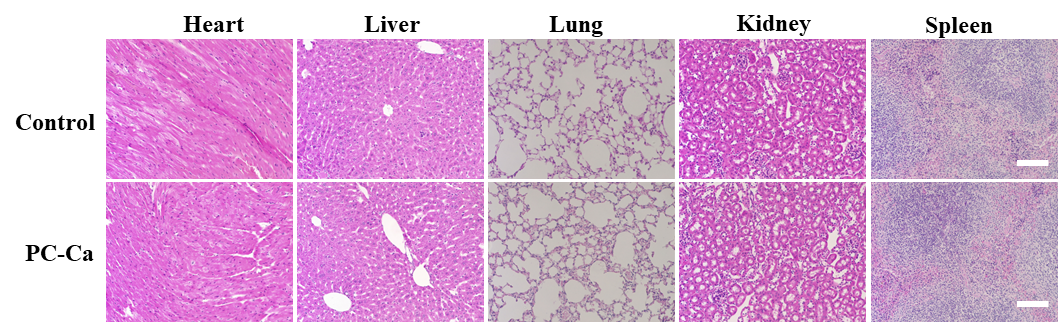


**Figure S5.** Representative hematoxylin and eosin (H&E) staining of heart, liver, lung, spleen and kidney tissues in Control group and PC-Ca group.


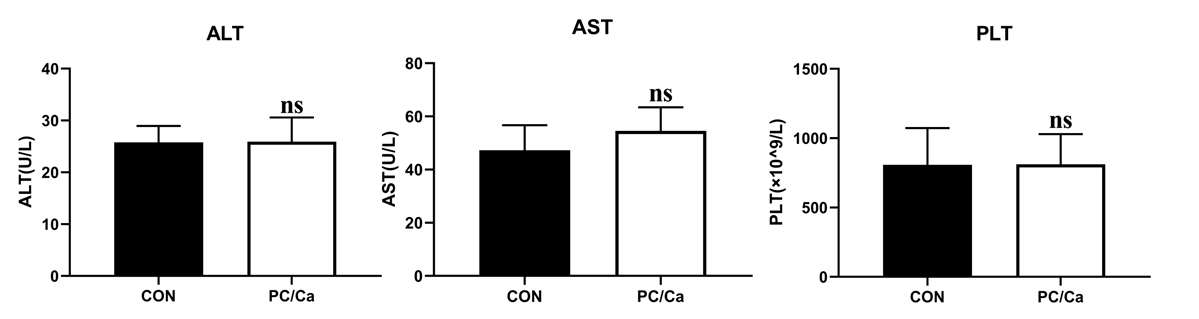


**Figure S6.** The levels of ALT, AST and PLT in Control group and PC-Ca group.


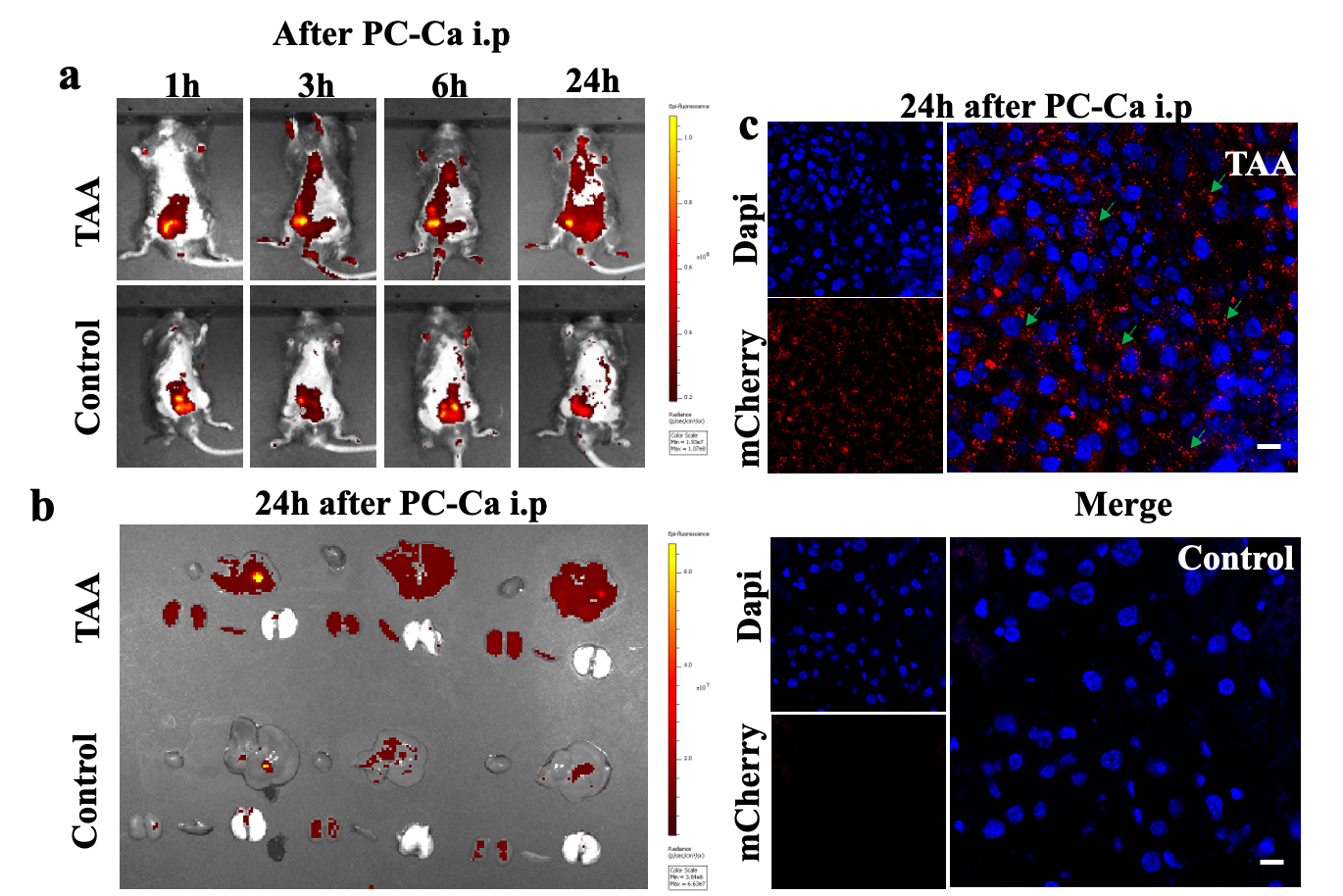


**Figure S7.** In vivo biodistribution of PC-Ca Capsules after intraperitoneal injection. (a) In vivo fluorescence images of mice after injecting PC-Ca at 1h, 3h, 6h, 24h. (b) Ex vivo fluorescence of PC-Ca in the main organs at 24 h after injection. (c) the distribution of mCherry-PC-Ca in liver tissue sections was observed by confocal laser scanning microscopy. (Red represents mCherry, Blue represents cell nucleus, Green arrows represent PC-Ca. Scale bar＝20μm.


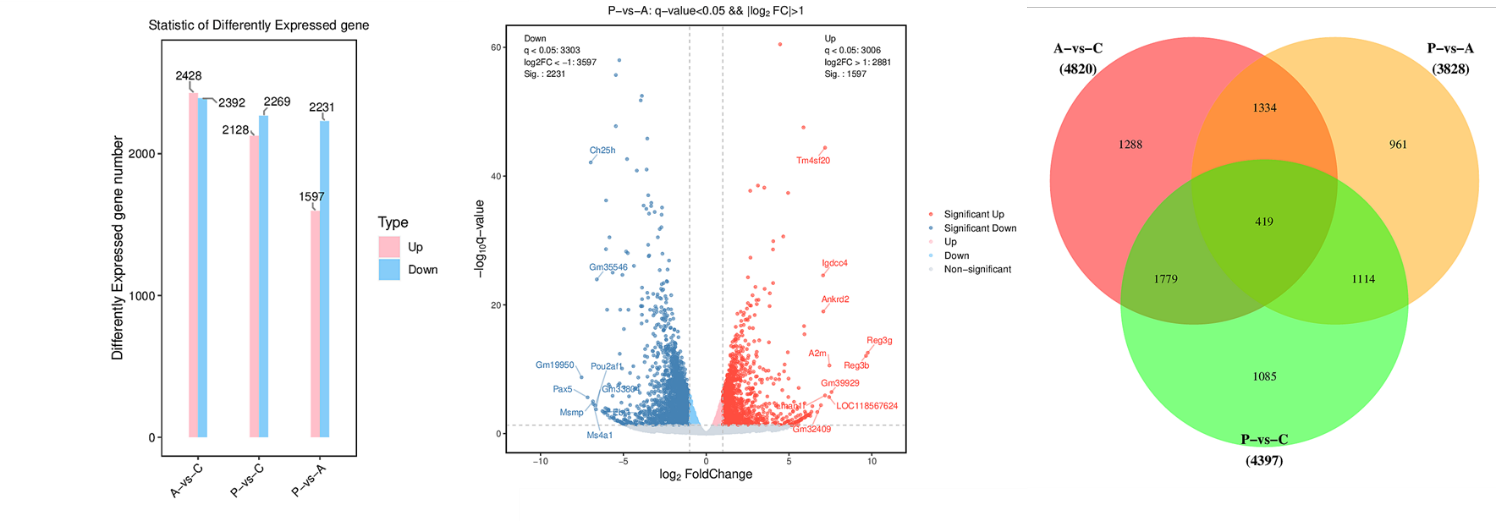


**Figure S8.** Correlations and principal component analyses (PCA) of the whole-genome expression profiles in control, ALF, PC-Ca group. (C: Control group, A: ALF group, P: PC-Ca group)


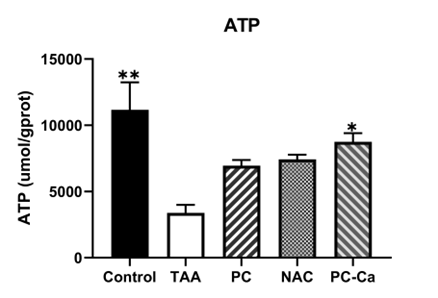


**Figure S9.** ATP production of liver tissues in each group.

| **Table S1.** Primers used in qRT-PCR to evaluate gene expression. | | |
| --- | --- | --- |
| **Gene** | **Forward primer sequence** | **Reverse primer sequence** |
| mos- MLKL | GGTTGAAGAATCCACGGACAAGAAG | AAAGCAAGCAAGCAAGGGAGAAAG |
| mos-RIPK1 | CGCCTACACCAGCCACAGTC | GAAGTGCCAATGAGTGCCAGAATG |
| mos- RIPK3 | GACACGGCACTCCTTGGTATCC | TTGAGGCAGTAGTTCTTGGTGGTG |
| mos-IL-6 | CTTCTTGGGACTGATGCTGGTGAC | TCTGTTGGGAGTGGTATCCTCTGTG |
| mos-TNF-α | GGACTAGCCAGGAGGGAGAACAG | GCCAGTGAGTGAAAGGGACAGAAC |
| mos-IL-1β | CACTACAGGCTCCGAGATGAACAAC | TGTCGTTGCTTGGTTCTCCTTGTAC |
| mos-GAPDH | GGCAAATTCAACGGCACAGTCAAG | TCGCTCCTGGAAGATGGTGATGG |
